# Les accidents de la vie courante chez l’enfant à Dakar: à propos de 201 cas

**DOI:** 10.11604/pamj.2017.27.272.6759

**Published:** 2017-08-10

**Authors:** Azhar Salim Mohamed, Aloïse Sagna, Mbaye Fall, Ndeye Aby Ndoye, Papa Alassane Mbaye, Aimé Lakh Fall, Alou Diaby, Oumar Ndour, Gabriel Ngom

**Affiliations:** 1 Centre de Santé des HLM de Dakar, Sénégal; 2 Service de Chirurgie Pédiatrique, Hôpital d’Enfants Albert Royer de Dakar, Sénégal; 3 Service de Chirurgie Pédiatrique, Centre Hospitalier Universitaire (CHU) Aristide Le Dantec de Dakar, Sénégal; 4 Institut de Formation et de Recherche en Population, Développement et Santé de la Reproduction (IPDSR), UCAD, Dakar, Sénégal Albert Royer de Dakar, Sénégal

**Keywords:** Accidents vie courante, enfant, accident domestique, chute, fractures, Accidents of the everyday life, child, domestic accident, fall, fractures

## Abstract

Les accidents de la vie courante (AcVC) sont fréquents chez l’enfant et peuvent être à l’origine de lésions handicapantes et de décès. L’objectif de notre travail était d’étudier les aspects épidémiologiques et lésionnels des AcVC à Dakar. C’est une étude transversale descriptive menée du 1^er^ Janvier 2013 au 30 juin 2013. Les enfants victimes d’accidents domestiques, d’accidents de sport et de loisirs ou d’accidents scolaires ont été inclus. Nous avons étudié des paramètres généraux et des paramètres ayant trait à chaque type d’AcVC. Deux cent et un enfants ont été inclus, ce qui représentait 27% des consultations aux urgences. Il y avait 148 garçons et 53 filles. Les enfants de moins de 5 ans étaient les plus touchés (37,8%). Le football et le jeu de lutte étaient les grands pourvoyeurs d’AcVC. Les AcVC survenaient principalement à domicile (58,2%) et dans les aires de sport et de loisirs (31,8%). Les fractures prédominaient dans les différents types d’AcVC: 54,9% des accidents domestiques, 68,8% des accidents de sport et de loisirs et 40% des accidents scolaires. Au plan épidémiologique, nos résultats sont superposables à la littérature. Les fractures prédominent à l’opposé de la littérature où les contusions sont prépondérantes. Le jeu de lutte est le plus grand pourvoyeur de ces fractures après le football. La connaissance des aspects épidémiologiques et lésionnels permet de mener des campagnes de prévention des AcVC à Dakar.

## Abstract (en)

Accidents of everyday life (AcVC) are common in children and can led to disabling injuries and death. This study aimed to analyze the epidemiological aspects of AcVC and the related injury mechanisms in Dakar. We conducted a descriptive, cross-sectional study conducted from 1 January 2013 to 30 June 2013. All the children victims of domestic accidents, sport and leisure accidents or school accidents were included. We studied some general parameters and some parameters related to each type of AcVC. Two hundred and one children were included, accounting for 27% of emergency consultations. There were 148 boys and 53 girls. Children less than 5 years of age were most affected (37.8%). Football and wrestling game were the main causes of AcVC. AcVC occur mainly at home (58.2%) and in the areas of sport and recreation (31.8%). The fractures predominated in the different types of AcVC: 54.9% of domestic accidents, 68.8% of sport and recreation accidents and 40% of school accidents. From an epidemiological perspective, our results are superimposable to literature. Fractures predominated contrary to literature where bruises were preponderant. Wrestling game is the main cause of these fractures, after football. The acquisition of knowledge about the epidemiological aspects of AcVC and the related injury mechanisms will allow for prevention campaigns in Dakar.

## Introduction

Un accident de la vie courante (AcVC) est définit comme un traumatisme non intentionnel survenant à la maison ou dans ses abords immédiats (accidents domestiques), sur les aires de sport et de loisirs (accidents de sport et de loisirs) et à l’école (accidents scolaires), à l’exception des suicides, des agressions et des accidents de la circulation et du travail [1]. Les enfants sont souvent victimes d’AcVC. On estime que près de 40 % des AcVC, tous âges confondus, surviennent chez les enfants davantage entre 1 et 4 ans et entre 10 et 14 ans [1], alors que les traumatismes pédiatriques ne représentent que 14 % de la pathologie traumatique globale [2]. Les accidents domestiques sont les plus fréquents [3, 4]. La chute est le mécanisme prédominant suivie des coups et collisions et des écrasements [4]. Les AcVC chez les enfants peuvent provoquer diverses lésions comme les contusions, les plaies, les fractures, les luxations et les entorses [4]. Les AcVC entraînent parfois des séquelles chez l’enfant et de sérieuses répercussions financières pour la société. Ils tuent plus que les accidents de la route [5]. Dans les pays de l’Union européenne, 120 000 décès sont dus aux AcVC chaque année et plusieurs millions de décès sont répertoriés dans le monde [6]. Dans le contexte africain dominé par les pathologies infectieuses et les carences nutritionnelles, la pathologie accidentelle demeure un réel problème, mais elle est malheureusement peu documentée [7]. Au Sénégal, l’absence de données sur les AcVC chez l’enfant, nous a poussé à réaliser cette étude dont le but est de rapporter les aspects épidémiologiques et lésionnels des AcVC dans un Hôpital Universitaire de Dakar.

## Méthodes

Nous avons réalisé une étude transversale de type descriptif sur les AcVC pris en charge au niveau de l’unité des urgences du service de Chirurgie Pédiatrique du CHU Aristide Le Dantec de Dakar. L’étude s’est déroulée sur une période de six mois allant du 1er janvier 2013 au 30 juin 2013. Tous les patients âgés de moins de 16 ans ayant présenté un accident non intentionnel survenant à domicile ou dans ses abords immédiats, sur les aires de sport et de loisirs et à l’école ont été inclus dans l’étude. Les patients reçus pour un accident de la circulation, un accident de travail, un suicide et une agression ont été exclus de l’étude. Nous avons étudié des paramètres généraux et des paramètres ayant trait aux différents types d’AcVC. Les paramètres généraux comportaient la fréquence des AcVC par rapport à l’ensemble des consultations faites au niveau de l’unité des urgences, l’âge des enfants exprimé en groupes (0-5 ans, 6-10 ans et 11-15 ans), le sexe, le rang occupé par l’enfant dans la lignée maternelle, le niveau socio-économique des parents, le jour et l’heure de survenue de l’accident, et le lieu de survenue de l’accident qui permettait d’identifier les accidents domestiques, les accidents scolaires et les accidents de sport et de loisirs. Nous avons ensuite reparti les enfants selon le type d’AcVC. Ainsi pour les accidents domestiques nous avons tenu compte de l’âge et du sexe des enfants, du lieu de survenue de l’accident, du mécanisme, du type de lésion occasionnée et du siège de cette lésion. Les mêmes paramètres ont été étudiés pour les accidents scolaires et les accidents de sport et de loisirs, en y ajoutant le type de jeu ou de sport pratiqués au moment de l’accident. La collecte des données a été faite à partir d’une fiche d’enquête préétablie. La saisie des données a été effectuée avec le logiciel SPSS 18 et Microsoft Office (Word & Excel) 2007.

## Résultats

Deux cent et un cas d’AcVC étaient recensés durant notre période d’étude, ce qui représentait 27 % de l’ensemble des consultations aux urgences chirurgicales pédiatriques du CHU Aristide Le Dantec durant la même période. Sur les 201 enfants enregistrés, 73,6 % étaient des garçons et 26,4 % étaient des filles soit un sexe ratio de 2,8. La tranche d’âge la plus touchée était comprise entre 0 et 5 ans avec 37,8 %, suivie des grands enfants de 11 à 15 ans avec 32,3 % puis des enfants de 6 à 10 ans avec 29,9 %. Ce sont les aînés qui étaient les plus touchés avec 31,5 % des cas (Tableau 1). La majorité des familles des enfants ayant présenté un AcVC étaient de niveau socio-économique moyen (60,2 %). Elles étaient suivies des familles de bas niveau socio-économique (27,9 %,) puis de niveau socio-économique bon (11,9 %). Le mercredi était le jour d’affluence maximale (20,4 %) suivi du dimanche (14,4%). Le mardi, le jeudi et le samedi suivaient respectivement avec 12,9 % des cas chacun. Onze virgule neuf pour cent des AcVC survenaient le lundi. Près de la moitié des AcVC survenait le soir entre 16 et 18 heures (42,3 %). Les autres accidents étaient enregistrés entre 13 et 15 heures (21,9 %), entre 6 et 12 heures (20,4 %) et entre 19 et 00 heure (15,4 %). C’est à la maison que l’on trouvait le plus d’AcVC avec 58,2 % des cas. Elle était suivie des aires de sport et de loisirs (31,8 %) et du milieu scolaire (10 %).

**Tableau 1:** Effectif des AcVC enfonction du rang de l’enfant

Rang de l’enfant	Effectif	Pourcentage (%)
1	56	31,5
2	45	25,3
3	25	14
4	20	11,2
5	10	5,6
6	10	5,6
7	6	3,4
8	3	1,7
9	3	1,7
**Total**	**178**	**100**

### Les accidents domestiques

Les garçons étaient plus touchés avec un sexe ratio de 1,8. La moitié des accidents domestiques survenait avant l’âge de 5 ans (53 %). Les enfants âgés entre 6 et 10 ans représentaient 33,3 % des cas et ceux âgés de 11 à 15 ans faisaient 13,7 % des cas. Près du tiers des accidents domestiques (36,8 %) ont eu lieu dans les abords immédiats du domicile (la cour et le jardin). A l’intérieur de la maison, c’est dans la salle de séjour que se sont produits le plus d’accidents (22,2 %), suivie des escaliers (16,2 %) et de la cuisine (12,8 %). La chute était impliquée dans 63,2 % des cas. Les brûlures venaient en deuxième position avec 13,7 % des cas suivies des écrasements (11,1 %), des collisions (5,1 %), des ingestions de corps étrangers (3,4 %). Les autres mécanismes rares (morsures, blessure par objets tranchants…) représentaient 3,5 % des cas. La fracture était retrouvée chez la moitié des enfants victimes d’accidents domestiques avec 54,9 % des cas. La brûlure occupait la deuxième place avec 14,2 % des cas suivie des plaies (12,4 %) puis venaient ensuite les contusions (8 %), les entorses (1,8 %) et les luxations (1,8 %). Les lésions concernaient surtout les membres supérieurs (58,9 %) et inférieurs (27,1 %). Les lésions à siège multiple (8,4 %), la tête (3,8 %) et le tronc (1,8 %) suivaient respectivement.

### Les accidents de sport et loisirs

**Figure 1:**
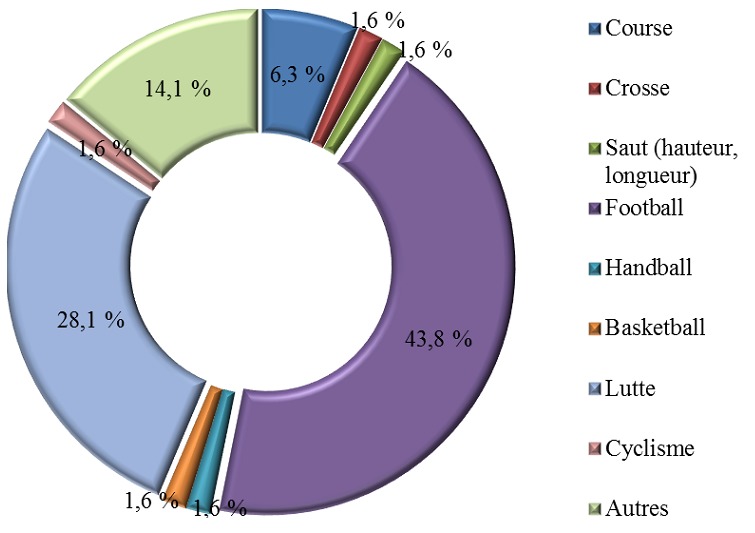
Répartition des accidents de sport et de loisir en fonction du type de sport

Il y avait 58 garçons et 6 filles soit un sexe ratio de 9,6. Les enfants de plus de 10 ans étaient les plus concernés par les accidents de sport et de loisirs (53,1 %) suivis des enfants de 6 à 10 ans (28,1 %) puis des enfants de moins de 5 ans (18,8 %). Près de la moitié des enfants victimes d’accidents de sport et de loisirs jouaient au football (43,8 %). La lutte était la deuxième circonstance de survenue d’accidents de sport et de loisirs (28,1 %). Les détails sont représentés dans la Figure 1. La chute était retrouvée dans 43 cas soit dans 67,2 % des cas, suivie des chocs avec 32,8 % des cas. Sur les 64 cas d’accidents de sport et de loisirs, nous avons dénombré 44 fractures représentant 68,8 % des cas, puis venaient les contusions (12,5 %), les luxations (6,3 %), les plaies (4,7 %) et les entorses (1,6 %). Les membres étaient le siège de prédilection des traumatismes lors des accidents de sport et de loisirs (95,1 %) suivis du tronc (3,3 %) puis de la tête (1,6 %).

### Les accidents scolaires

**Figure 2:**
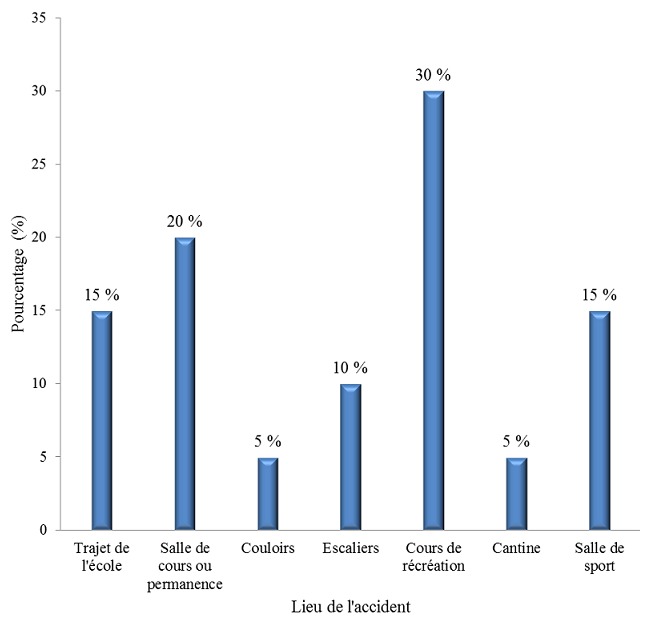
Les accidents scolaires selon le lieu de survenue

Il y avait 14 garçons et 6 filles soit un sexe ratio de 2,3. La tranche d’âge de 11 à 15 ans était la plus concernée suivie des enfants âgés entre 6 et 10 ans (15 %) et des enfants de moins de 5 ans (10 %). La majorité des cas (30 %) a eu lieu à la cour de récréation et 20 % des cas survenaient à la salle de cours ou de permanence. Quinze pour cent des accidents scolaires survenaient sur le trajet de l’école (Figure 2). La chute était responsable de l’accident scolaire dans 17 cas soit dans 85 % des cas et la collision dans 3 cas soit dans 15 % des cas. Les fractures et les contusions étaient prédominantes avec respectivement 40 % et 30 % des cas, suivies des entorses (15 %) et des luxations (5 %). Les membres supérieurs étaient essentiellement concernés, soit dans 66,7 % des cas, suivis des membres inférieurs (27,8 %). Les lésions à siège multiple représentaient 5,6 % des cas.

## Discussion

Les AcVC représentent un peu plus du quart de l’ensemble des consultations à l’unité des urgences du service de Chirurgie Pédiatrique du CHU A. Le Dantec pendant notre période d’étude. Ce résultat confirme les données de plusieurs séries de la littérature [1, 8–15]. Nous constatons dans notre étude une prédominance masculine, comme dans la majorité des séries de la littérature [1, 4, 8–15]. Cette prédominance masculine pourrait être liée à un comportement plus agressif des garçons, volontiers plus rétifs quant aux interdits [10]. Cependant, dans notre étude, il existe une surreprésentation masculine par rapport aux études comparatives. Cet aspect peut être lié au fait que, dans notre contexte, les filles sont souvent confinées dans les tâches ménagères à la maison, ce qui explique, dans une certaine mesure, leur faible représentation. Dans notre étude, c’est la tranche d’âge de 0 à 5 ans qui est la plus touchée. Ce résultat concorde avec la majorité des séries de la littérature [9, 10, 12, 13, 16]. La place des enfants âgés de 6 à 10 ans et de 11 à 15 ans est variable selon les études. La répartition des tranches d’âge selon diverses études est représentée sur le Tableau 2. Dans notre étude, ce sont les aînés qui sont les plus touchés avec 31,5 % des cas. Dans 70,8 % des cas, l’enfant a un rang inférieur ou égal à 3. Ce résultat est confirmé par les travaux d’Ategbo au Gabon avec 71 % [10] et ceux de l’Institut de Veille Sanitaire à Saint-Maurice avec 93,5 % [8]. Cela pourrait être lié au fait que les grands enfants sont moins surveillés dans les familles nombreuses où une plus grande attention est portée aux tout petits. Ategbo a donné une explication similaire [10]. La majorité des familles des enfants de notre étude est d’un niveau socio-économique moyen et bas. Cela concorde avec des études de la littérature [10, 16, 17]. Il existe, en effet, un lien entre le statut socio-économique et certains risques d’exposition aux AcVC telles que les conditions d’habitation [16, 17] et une absence d’information pour l’acquisition de connaissances utiles dans la prévention des accidents. Il est ainsi important de tenir compte des facteurs socio-économiques lors de l´élaboration d´un programme d´intervention [13]. Les mercredis sont les jours d’affluence maximale dans notre service. Les autres jours semblent plus calmes. Nous n’avons pas trouvé d’explication à ce phénomène. Reboli en France [9] a trouvé un maximum de cas pendant les jours ouvrables tandis qu’Ategbo au Gabon [10] retrouvait une prédominance des jours de week-end. Selon Ategbo, ce résultat pourrait s’expliquer par la présence au domicile, les samedi soir et dimanche, des enfants qui sont moins surveillés car il s’agit des jours de repos parental. Dans notre série, près de la moitié des AcVC surviennent en fin d’après-midi entre 16 et 18 heures, tranche horaire correspondant à la descente des enfants de l’école. Ils jouent dans la rue et se livrent à des activités de jeux ou de sport. Selon les résultats du réseau Epac en 2004 [1], un maximum de cas est noté en fin d’après midi jusqu’à 22 heures. Cette étude a démontré que la chronologie des AcVC sur la journée correspond approximativement à celle des activités générales avec un maximum entre 16 et 19 heures [1]. Dans notre étude, c’est à la maison que l’on trouve le plus d’AcVC, lieu suivi des aires de sport et de loisirs et du milieu scolaire. Nos résultats concordent avec plusieurs séries de la littérature [3, 4, 11, 12]. Par contre, les accidents en milieu scolaire occupaient la deuxième position dans les séries françaises de Reboli [9] et de l’Epac [1].

**Tableau 2:** Répartition des AcVC selon les tranches d’âge

Etude	Pays	0 – 5 ans	6 – 10 ans	11 – 15 ans
Epac [1]	France	37,1 %	25,6 %	37,4%
Zidouni [16]	Algérie	58,3 %	27,8 %	13,8 %
Phelan [12]	Etats-Unis	43 %	23,5 %	17,5 %
Notre étude	Sénégal	37,8 %	29,9 %	32,3 %

### Les accidents domestiques

Notre étude retrouve une prédominance masculine ainsi qu’une fréquence plus importante chez les enfants âgés de moins de 5 ans. Ces résultats étaient les mêmes que pour les études comparatives [4, 10, 12–14, 17, 18–20]. Dans l’étude de Thélot [3], trois AcVC sur quatre avant l’âge de 2 ans survenaient à domicile. Après l’âge d’un an, les enfants marchent, ce qui leur permet de découvrir le monde qui les entoure, de jouer à l’extérieur, et d’échapper plus facilement à la surveillance de leurs parents [1]. Le lieu de survenue le plus fréquent dans notre série est la cour, lieu suivi de la salle de séjour (salon), des escaliers, puis de la cuisine. Ategbo au Gabon a retrouvé la cour suivie de la chambre, puis de la cuisine [10]. Zidouni en Algérie a fait un constat différent avec une prédominance des accidents domestiques dans la chambre suivie de la cour, de la cuisine puis des escaliers [16] rejoignant des données françaises [1, 4, 9]. Ce constat pourrait s’expliquer par le fait que dans notre contexte, en Afrique Noire, les enfants passent plus de temps à jouer dans la cour de la maison. Dans notre étude, la chute constitue le principal mécanisme de l’accident domestique, suivie des brûlures, puis des écrasements. Cela peut être expliqué par le fait que le jeu est la principale activité des enfants à domicile, et que celui-ci est constitué de courses et de sauts pouvant occasionner ces chutes [10]. D’autres études [1, 4, 9, 12, 13, 16, 19] ont placé également la chute comme le principal mécanisme de l’accident domestique. Les principales lésions sont les fractures, alors que dans les études occidentales, les contusions et les plaies viennent en première position [1, 4, 9, 12, 19]. Le football et la lutte sont à l’origine de beaucoup de lésions fracturaires [11], ce qui explique la prédominance de ces lésions dans notre étude. Dans la majorité des cas, les membres sont la principale localisation des lésions lors d’un accident domestique. Ce résultat est en conformité avec les données de la littérature [1,12,^16^]. D’autres auteurs ont retenu la tête comme la principale cible des traumatismes surtout chez les jeunes enfants avant 5 ans [9, 11, 15, 18]. Dans notre étude il y a un biais de sélection car les traumatismes de la tête sont pris en charge presqu’exclusivement dans les services de Neurochirurgie.

### Les accidents de sport et loisir

Nous notons une surreprésentation masculine dans notre série avec un sexe ratio de 9,6. Ce résultat concorde avec les données de la littérature [1, 3, 4, 9, 21]. Cela est lié au fait que les garçons prennent plus de risques que les filles, qu’ils sont plus actifs et qu’ils agissent de façon plus impulsive [22]. De plus, au Sénégal, les filles restent à la maison aidant les mamans dans les tâches ménagères ce qui explique leur faible exposition aux accidents de sport et loisirs. Dans notre étude, les accidents de sport et de loisirs surviennent essentiellement chez le grand enfant entre 11 et 15 ans. Dans la série de Thélot [4], la proportion des accidents de sport et de loisirs augmentait avec l’âge et représentait un tiers des AcVC à partir de 12 ans. D’autres auteurs ont fait le même constat [1, 4, 9, 21, 23]. Dans notre série les jeux et sports incriminés sont surtout le football et la lutte. Le football est le sport le plus populaire dans le monde ; ce qui pourrait expliquer la prédominance de ce type de sport dans notre étude. Contrairement à toutes les études de la littérature, la lutte arrive en deuxième position dans notre étude. La lutte est un véritable phénomène de société au Sénégal où c’est le sport le plus populaire. Elle permet aux lutteurs de gagner rapidement beaucoup d’argent. Pour beaucoup d’enfants ces lutteurs représentent un modèle de réussite sociale. Les enfants essaient donc de les imiter pour leur ressembler un jour. Puisqu’ils ne maîtrisent pas les techniques de lutte, ils font beaucoup de fractures lors des chutes. Dans notre étude, les lésions dominantes sont les fractures de même que dans l’étude de Pelech [24]. Cependant, dans la majorité des séries de la littérature, on trouve une prédominance des contusions, suivies des entorses puis des fractures [1, 3, 4, 9, 13]. Cela s’explique, par le fait que, dans notre étude, le football et la lutte, grandes pourvoyeuses de fractures prédominent. On constate, dans notre série, que les lésions siègent principalement au niveau des membres, résultat comparable aux données de diverses études [1, 12, 24].

### Les accidents scolaires

Dans notre étude, ils représentent 10 % des AcVC. Cette fréquence est comparable avec les résultats obtenus par Thélot en 2010 en France [4] et Kieran en 2005 aux Etats-Unis [12]. Nous constatons une prédominance masculine dans notre étude avec un sexe ratio de 2,3. La tranche d’âge de 11 à 15 ans est la plus touchée dans notre étude. Selon Chapuis c’est parmi les élèves les plus âgés que l’on retrouve les incidences les plus élevées [25]. Dans notre étude, la cour de récréation constitue le lieu de prédilection des accidents scolaires. La cour de récréation est un lieu de détente, particulièrement propice aux jeux et aux sports car les enfants aiment les espaces. Dans notre série comme dans plusieurs études [1, 8, 9, 26, 27], la chute reste le principal mécanisme. Les fractures prédominent, suivies des contusions et des entorses. Dans diverses séries occidentales, les contusions occupaient la première place suivie des plaies puis venaient ensuite les fractures et les entorses [1, 3, 4, 8, 9, 26]. Cela s’explique, par le fait que, dans notre étude, le football et la lutte, qui sont à l’origine de beaucoup de fractures prédominent. Les membres sont les plus fréquemment atteints dans notre étude. Ce résultat concorde avec les données de la littérature [1, 3, 4, 8, 9, 12]. Lors d’une chute, l’enfant tombe avec une réception, le plus souvent, sur les membres.

## Conclusion

Les AcVC chez l’enfant sont fréquents à Dakar. Ils sont dominés par les accidents domestiques et touchent essentiellement le garçon âgé de moins de 5 ans, victime le plus souvent d’une chute. Les lésions sont dominées par les fractures qui siègent surtout aux membres. Nous n’avons pas enregistré de décès car les traumatismes sont de basse énergie.

### Etat des connaissances actuelles sur le sujet

- Les AcVC sont fréquents et le plus souvent mortels en Europe, 120 000 décès chaque année;
- Les enfants sont souvent victimes d’AcVC et d’avantage ceux âgés entre 1 et 5 ans;
- La chute représente le principal mécanisme mis en cause dans ces accidents qui sont le plus souvent à l’origine de contusions et de plaies.

### Contribution de notre étude à la connaissance

- Notre étude confirme la grande fréquence des AcVC chez les petits enfants et la prédominance masculine;
- Dans notre contexte, les fractures étaient la lésion la plus fréquente;
- Notre étude se veut le prélude d’autres études de grande envergure permettant de faire d’utiles recommandations pour la prévention des AcVC à l’échelle nationale.

## Conflits d’intérêts

Les auteurs ne déclarent aucun conflit d’intérêts.
